# Ionizing Radiation: Effective Physical Agents for Economic Crop Seed Priming and the Underlying Physiological Mechanisms

**DOI:** 10.3390/ijms232315212

**Published:** 2022-12-02

**Authors:** Jiaqi Wang, Yixin Zhang, Libin Zhou, Fu Yang, Jingpeng Li, Yan Du, Ruiyuan Liu, Wenjian Li, Lixia Yu

**Affiliations:** 1 Institute of Modern Physics, Chinese Academy of Sciences, Lanzhou 730099, China; 2 College of Life Sciences, University of Chinese Academy of Sciences, Beijing 100049, China; 3 School of Biological Sciences, The University of Edinburgh, 57 George Square, Edinburgh EH89JU, UK; 4 Northeast Institute of Geography and Agroecology, Chinese Academy of Sciences, Changchun 130102, China

**Keywords:** ionizing radiation, seed priming, physiological mechanism, agricultural production, future needs

## Abstract

To overcome various factors that limit crop production and to meet the growing demand for food by the increasing world population. Seed priming technology has been proposed, and it is considered to be a promising strategy for agricultural sciences and food technology. This technology helps to curtail the germination time, increase the seed vigor, improve the seedling establishment, and enhance the stress tolerance, all of which are conducive to improving the crop yield. Meanwhile, it can be used to reduce seed infection for better physiological or phytosanitary quality. Compared to conventional methods, such as the use of water or chemical-based agents, X-rays, gamma rays, electron beams, proton beams, and heavy ion beams have emerged as promising physics strategies for seed priming as they are time-saving, more effective, environmentally friendly, and there is a greater certainty for yield improvement. Ionizing radiation (IR) has certain biological advantages over other seed priming methods since it generates charged ions while penetrating through the target organisms, and it has enough energy to cause biological effects. However, before the wide utilization of ionizing priming methods in agriculture, extensive research is needed to explore their effects on seed priming and to focus on the underlying mechanism of them. Overall, this review aims to highlight the current understanding of ionizing priming methods and their applicability for promoting agroecological resilience and meeting the challenges of food crises nowadays.

## 1. Introduction

Food security, as a pressing development issue, has attracted the attention of many countries [1,2]. As the “chip” of agriculture, seed quality is the foundation of food security, and it is a crucial indicator for a healthy agricultural production system. Seed germination is a highly regarded trait that determines the crop stand and performance. However, throughout the plant’s life cycle, the germination stage is the most sensitive period to the environment, and it is vulnerable to a series of internal and external restrictive factors that lead to the physiological activities slowing down, growth retardation, and stagnation. According to the statistics, 90% of the edible crops are cultivated from seeds, and insufficient seed germination and poor seedling growth often result in huge crop losses. Therefore, the seed quality improving is a primary way to ensure food security [3,4,5].

**Figure 1:**
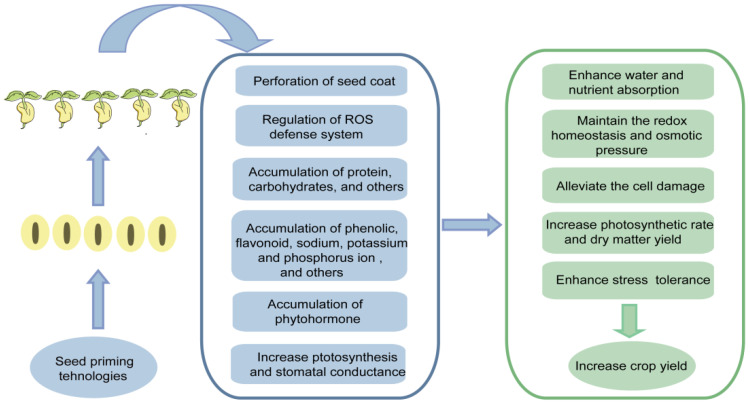
A schematic diagram describing the positive effect of seed priming technologies.

Seed germination, as the first stage of the plant’s development, is a delicate process with strict requirements, for instance, the seeds with poor embryonic development, serious empty shells, or lignified shells are often hard to germinate [6,7,8,9]. In addition, the extreme changes in the environmental conditions, such as salinity, temperature, heat, light, pH, and moisture, also pose devastating abiotic stresses for the seed germination and crop yield. Therefore, it is of great importance to develop new techniques for improving the seed germination and seedling growth. Seed priming is one of the primitive strategies applied to enhance early seed emergence and accelerate several processes involved in germination [10]. Under normal and stress conditions, seed priming techniques could be used to improve the seed quality, which is attributed to fast germination and more seedling vigor. As shown in Figure 1, some artificial seed priming methods can directly etch the seed husk, increase the seed coat permeability for water and other nutrition species, accelerate the hydration process between the cell wall and the protoplasm, initiate the metabolic processes to accumulate protein, carbohydrates, phenolics, flavonoids, phytohormones and others, and increase photosynthesis and stomatal conductance, consequently, improving the seed’s performance and crop yield. Further, the upregulation of the antioxidant defense system comprising enzymatic antioxidants, for instance, superoxide dismutase (SOD), peroxidase (POD), and catalase (CAT), as well as non-enzymatic antioxidants, such as glutathione (GSH) and ascorbate (AsA), is also beneficial for seed priming and seedling establishment, and this helps the seed to cope with stress coming from the environment [10,11,12,13,14].

**Figure 2:**
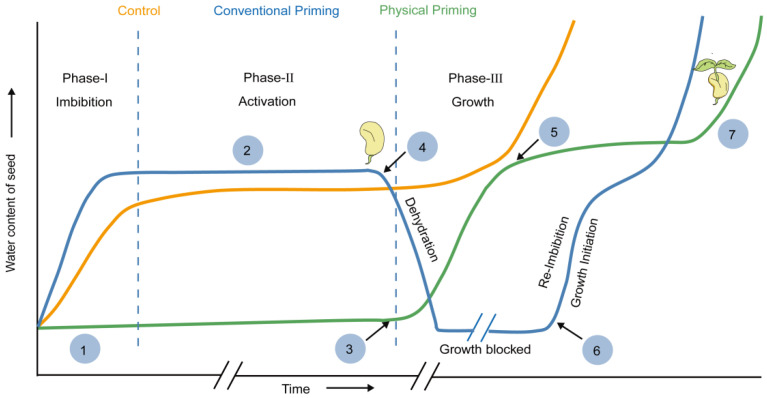
Schematic representation of the water uptake within the seed during germination in control, conventionally primed, and physically primed seeds. There are three stages in seed germination process: the first phase (imbibition phase) starts with the absorption of water, the phase II (metabolic activation) involves the activation of the enzymes, and this dramatically increases respiration and metabolism, and phase III (radical protrusion phase) mainly involves a process which regulates the cell elongation and the roots and hypocotyls protuberance from the seeds. The seed priming strategies principally occur during phase II, which stimulates the uptake of water and accelerates the pregerminative metabolic process. The yellow line represents a standard (control) germination process. The blue line corresponds to a conventional primed seed germination process. The green line (hypothetical) represents germination in the physical primed process. The figure is adapted from [3,10]. ① Initiation of seed priming. ② Energy metabolism, regulation of oxidative status, DNA repair, cell cycle activation, food reserve mobilization, alteration of hormonal status, de novo synthesis of mRNA for metabolic genes, synthesis of HSPs which probably protect cell proteins from damage and free radical scavenging enzymes which possibly protects the cell from damage due to lipid peroxidation. ③ Imbibition. ④ Denouement of conventional priming followed by drying. ⑤ Rapid cell division and elongation. ⑥ Storage of conventional primed seed. ⑦ Final germination.

As shown in Figure 2, there are three stages in seed germination. The first phase (the imbibition phase) starts with the absorption of water, the phase Ⅱ (metabolic activation) involves the activation of the enzymes and the dramatic increase in respiration and metabolism, while phase Ⅲ (the radical protrusion phase) involves the process which mainly regulates the cell elongation and the radicle protrusion. The seed priming strategies principally take place during phase Ⅱ, which stimulate the uptake of water and accelerate the pregerminative metabolic process [3]. The seed priming technique can date back to the ancient Greeks; Theophrastus (371–287 B.C.) found that soaking cucumber seeds in water for a pre-sowing treatment could improve their germination (Theophrastus, Enquiry into Plants, Book VII, I.6) [15]. In 1963, Ells noticed that tomato seeds treated with a nutrient solution showed an accelerated germination rate, which subsequently highlighted the key points in the seed treatment, and they proposed the modern concept of seed priming [16]. In the last couple of decades, different kinds of priming techniques have become more and more popular in agricultural production, i.e., hydropriming, halopriming, osmo-priming, magnetopriming, solid matrix priming, biopriming, nutripriming, antioxidants, phytohormones, plant growth regulators, and water and chemical priming [17,18,19,20,21,22,23]. However, these traditional seed priming methods have obvious disadvantages, such as a heavy workload, being inefficient and costly, having deleterious effects on the post-treatment storability, and always being unfriendly to the environment and food safety. Therefore, scientists introduced a low-dose physical agent, namely, ionizing radiation (IR). As an advanced and promising strategy for seed initiation and seedling formation, it could not only significantly promote seed germination, enhance seedling establishment and resilience under stress, but it could also show the advantage of being more efficient and environmental friendly [24,25,26,27].

**Figure 3:**
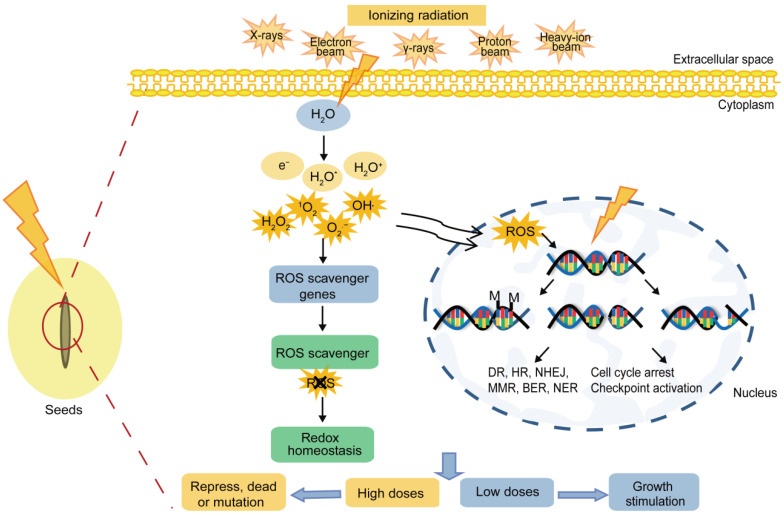
Schematic diagram of ionizing radiation acting on the organism. They can direct break the chemical bonds between atoms or molecules on the DNA molecule, causing the DNA single- or double-strand break, base deletion, or DNA methylation, and this can trigger cell cycle arrest and checkpoint activation, but most of the damage can be repaired correctly by direct repair (DR), homologous recombination (HR), non-homologous end joining (NHEJ), mismatch repair (MMR), base-excision repair (BER), or nucleotide excision repair (NER) mechanisms. However, the repair of the errors sometimes will be reserved and lead to chromosomal aberrations and genetic mutations. Further, in the way of the indirect actions, reactive oxygen species (ROS), namely, some strong oxidants and highly reactive free radicals, were also generated by water radiolysis, including short-life species, such as superoxide anion radical (O_2_^•−^), hydroxyl radical (OH^•^), singlet oxygen (^1^O_2_), and long-life species, such as hydrogen peroxide (H_2_O_2_), etc. These free radicals induced “oxidative stress” in cells, including lipid peroxidation and the oxidative modification of the proteins and nucleic acids, meanwhile, the content of antioxidants are also increased to regulate redox homeostasis. Excited water molecule (H_2_O*). Methylation (M).

**Figure 4:**
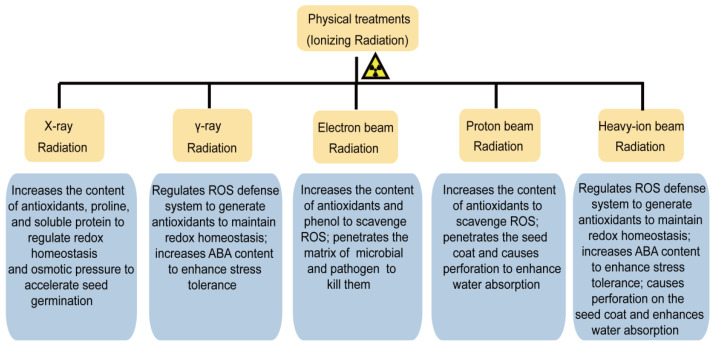
X-ray and γ-ray, electron beams, proton ions, and heavy-ion beams of physical agents used for seed priming and their mode of action.

Radiation is broadly divided into two categories: IR and non-ionizing radiation, and the latter only causes vibrations, rotations of the molecules, or changes in the state of the electron energy levels. On the contrary, the former one with a short wavelength (<100 nm) as well as higher energy and frequency, is mostly invasive, and it can ionize material molecules to converse neutral atoms or molecules into ions and provide energy to the electrons in a molecule to overcome the potential “nuclear attraction” barrier. It is adequate for knocking out electrons from atoms and generating charged ions, consequently triggering biological effects [28]. The main types of IR priming agents and their characteristics have been summarized in Table 1. Actually, IR inevitably exists in the environment because of the natural presence and human activities. IR mainly acts with biomolecules directly and indirectly, as shown in Figure 3. In the direct actions, it can break the chemical bonds between the atoms or molecules on the DNA molecule, causing the DNA single- or double-strand to break, base deletion, or DNA methylation and it can trigger cell cycle arrest and checkpoint activation. In the way of indirect action, the irradiation produces abundant reactive oxygen species (ROS) via water radiolysis, namely, some strong oxidants and highly reactive free radicals, including short-life species, such as superoxide anion radical (O_2_^•−^), hydroxyl radical (OH^•^), singlet oxygen (^1^O_2_), and long-life species, such as hydrogen peroxide (H_2_O_2_), etc. These free radicals induce “oxidative stress” within the cells, such as lipid peroxidation and the oxidative modification of proteins and nucleic acids [28,29]. Most of the damage could be repaired correctly by direct repair (DR), homologous recombination (HR), non-homologous end joining (NHEJ), mismatch repair (MMR), base-excision repair (BER), or nucleotide excision repair (NER) mechanisms. However, sometimes errors might be made during the repair process which could lead to chromosomal aberrations and genetic mutation. According to the literature, DNA damage induces most of the death of the organisms, and a significant part of the DNA damage (70–80%) is caused by ROS during the radiolysis of water, and as well as this, only 20–30% of the damage is triggered by IR quanta targeting DNA molecules [30]. Recently, numerous works have evidenced that X-rays, gamma rays (γ-rays), electron beams, proton ions, and heavy-ion beams are fast, uniform, economic, effective, and eco-friendly approaches for stimulating seed germination (Figure 4). However, as a promising approach for priming, an understanding of the physiological responses or the molecular mechanisms of the physical agents priming approaches is still scant. The purpose of this paper is to discuss the possibility of using ionizing physical agents to enhance the seed performance, and thus, increase crop productivity. It also attempts to highlight our current comprehensive understanding of the mechanisms of seed priming through IR.

## 2. Ionizing Physical Priming and Plant Responses

Nowadays, the conflict between a continuously growing global population and the loss of food production due to changing weather and decreasing arable land has been a great threat for food security. Hence, it is urgent to develop appropriate and effective methods to improve seed germination, seedling growth, and stress resistance under sub-optimal conditions to increase the crop yield.

As a key driver of food security, the seed quality directly impacts the seedling growth and the final crop production. However, germination is vulnerable to external unnormal conditions, usually leading to germination time delays, uneven germination, or weak seedlings. Therefore, it is worthwhile to develop various technologies to increase the production of or to impart biotic/abiotic resistance to crops [31]. Seed priming is regarded as being a very effective and acceptable approach worldwide [32]. In recent years, the applications of ionizing physical agents in agriculture for accelerating seed initiation, improving the seedling quality, enhancing their stress tolerance and increasing the crop yields have attracted much attention, and these strategies could be essential supplements to the conventional methods. These physical agents play a dual role: using high doses of agents puts stress on the seeds, and this has a negative impact on the seed’s vigor, however, low doses cause positive effects which are opposite to those found at high doses, this phenomenon is known as ‘hormesis’ [33,34]. Furthermore, numerous studies show that the effects are also related to the plant’s characteristics (e.g., species, cultivar, stage of development, tissue architecture, and genome organization) and radiation features (e.g., quality and the duration of exposure). This paper reviews the major IRs as priming agents and clarifies how they affect the seed germination. These IR agents have been used for many plan’s improvements, such as *Lathyrus chrysanthus* Boiss, *Holoptelea integrifolia*, *Oroxylum indicum*, *Hibiscus esculentus* L., and *Brassica rapa* [35,36,37,38]. A summary of the plants’ responses upon seed priming with an ionizing physical treatment along with their intensity and the dosage rate of such an exposure is partly given in Table 2. 

### 2.1. Priming with X Radiation

The X-ray is an important IR which has the characteristics of a high frequency (30 PHz~300 Ehz), a short wavelength (1 pm~10 nm), and a high energy (120 eV to 120 keV). In 1898, Maldiney and Thouvenin reported the X-ray accelerated germination of seeds first. In 1906, Evler also observed stimulated growth effects in the seeds of beans (*Glycine max* (L.) Merr), radishes (*Raphanus sativus* L.), lettuce (*Lactuca sativa*), and squash (*Cucurbita moschata*). In 1910, Schmidt obtained larger plants than the control ones after studying irradiation-soaked pea seeds (*Pisum sativum* L.) [34,60]. However, relatively few works have been published focusing on the effects of X-ray irradiation on seed priming in recent years [61,62]. The existing research relating to the stimulation effect is most associated with accelerating the seed germination, enhancing the seedling growth, earlier flowering, and increasing the crop yields by exposing the seeds or plants to low doses of X-rays [37,41,63,64]. The positive effects of the low dose of X-rays have been reported in *Phaseolus vulgaris* by Arena, and according to their research, the seeds exposed to 0.3 and 10 Gy significantly stimulated leaf lamina growth at 1 Gy/min, and the over-production of phenolic compounds in the cells may be a natural defense mechanism of the photosynthetic apparatus against radiation [42]. In okra (*Hibiscus esculentus* L.), stimulation effects were also been found to have occurred in after they were irradiated with low doses of X-rays (0, 0.25, 0.5, 1.0, 2.5, and 5.0 Gy at 1.9 kGy/min), wherein the length and the fresh and dry weights of the shoot and roots were significantly increased, together with the accumulation of the total pigment, nonenzymatic (AsA, GSH, and anthocyanin) and enzymatic antioxidants (ascorbate peroxidase (APX), POD, CAT, and SOD) [37]. In addition, the research has revealed that 0.3, 10, 20, 50, and 100 Gy X-rays markedly improved the plants’ height, the number of leaves, and the plant leaf area of *Solanum lycopersicum* L., and they formed more compact plants [40]. Similarly, the optimum dose of X-rays to stimulate germination and seedling vigor was established in a study on *Coffea arabica* [41]. Although the percentage of the germination of the seeds was not promoted in any treatment, the increase in the toughness and the diameter of the corn’s roots (*Zea mays* L.) were observed in a pre-sowing treatment with X-rays [65], while in other studies, irradiation produced a more uniform distribution of seedling heights in barley, and also, it increased the first emergence counts in broccoli (*Brassica oleracea* L.) and parsnip (*Pastinaca sativa* L.) [39,64].

### 2.2. Priming with γ Radiation

γ-rays are strong electromagnetic waves are, with high energy levels from 10 keV (kilo electron volts) to several hundred keV and shorter wavelengths (<0.001 nm) than X-rays have. With excellent penetration abilities, they can easily penetrate through glass and plastics, but they can be effectively blocked with the proper thickness of concrete or lead plate. γ-rays usually interact with the atoms or molecules in the plant cells, and they produce free radicals to influence the essential cells’ components and affect the morphology, anatomy, biochemistry, and physiology differentially, and together, they accelerate the seed germination, stimulate seedling growth, increase the production of secondary metabolites, and alleviate the biotic or abiotic stress effects [13,44,66,67,68]. The positive impact of low-dose γ-rays has been reported by Akshatha, and compared to the controls, 25 and 200 Gy doses of γ-rays enhanced the rate of seed germination in *Holoptelea integrifolia* and *Oroxylum indicum*, meanwhile, in the case of *Terminalia chebula*, irradiation with a 25 Gy dose also produced the low-dose hormesis phenomenon [35]. In *Lathyrus chrysanthus* Boiss, a low dose of γ-rays (50 Gy at 0.8 kGy/h) stimulated an increased germination percentage, seedling and root lengths, fresh weight, dry matter content, and total chlorophyll content of the seedlings [36]. Additionally, in barley, 16–20 Gy γ-rays enhanced the contents of glucose-6-phosphate dehydrogenase, pyruvate kinase, and guaiacol peroxidase activity, which induced the accelerating pace of the seedling’s development [43]. With the optimization of the development parameters by 150 Gy γ-rays, the grain yield for *Zea mays* and *Arachis hypogaea* was markedly increased [69]. In addition to the above, the positive effects of γ-ray irradiation has also been observed in other crops, such as wheat (*Triticum aestivum* L.) [70], garden cress (*Lepidium sativum* L.), sesame (*Sesamum indicum* L.) [71], rice (*Oryza sativa* L.), and mung (*Phaseolus mungo* L.) [72].

It is well known that the stresses are always caused by droughts, salinity, heavy metals, heat, cold, and pathogenic bacteria, and they induce various negative impacts on the plant’s germination, growth, and development, for instance, reducing the activities of the metabolic enzymes, damaging the protein by over-producing ROS, as well as inhibiting the crop yield via retarding germination and hindering normal nutrition absorption [46,73,74]. The studies have shown that low doses of γ-rays could relieve the above stresses effectively. For example, a low dose of γ-rays (100 Gy) markedly alleviated the deterrent of salinity and drought stress by promoting the activities of CAT, SOD and APX, and increasing the proline contents which are conducive to the accumulation of dry matter [44]. The exposure of highland barley seeds to a 50 Gy γ-ray irradiation enhanced their stress tolerance to salinity, lead, and cadmium, and it was involved in reducing hydrogen peroxide (H_2_O_2_) and improving the chloroplasts ultrastructure [46,48]. The low dose of the γ-ray (50 Gy) treatment was also used to stimulate *Arabidopsis* seedlings’ tolerance to heat damage, and according to the study, it promoted the seedlings’ growth and enzymes’ activities, and as well as this, it upregulated the expression levels of the genes associated with resistance establishment [75]. Otherwise, in chilling-stressed coriander (*Coriandrum sativum* L.), γ-ray irradiation notably promoted the growth parameters and yield components, and it increased the content of ABA and soluble sugars, which are concomitant with the accumulation of photosynthetic pigments, carbohydrate contents, and antioxidants [76]. Although the final germination capacity was not changed, the γ-ray irradiation significantly reduced the percentage of fungal incidence in millet grains (*Pennisetum gluucum* L.) [45], and it avoided insects and microorganism growth in bean seeds [77].

### 2.3. Priming with Electron Beam Radiation

The electron beam, a stream of electrons generated by the bombardment of charged particles or strong electric fields, can be controlled by the switch of the electron accelerator. In recent years, a new type of technology, namely, the electron beam to target turning X-ray (EBTTX) was invented. It is a bremsstrahlung X-ray produced by the sudden deceleration of a high-energy electron beam bombarding heavy metals. This irradiation technology has the advantages of both the electron beam and X-ray irradiations, such as easy operation, high accuracy, no radioactive source, strong penetration ability, and good repeatability [60,78]. In the past few years, it has not only been used to sterilize the microbial contamination and combat the pathogenic on plants to prevent pre-harvest produce contamination, but it also has potentially accelerated the plants’ growth at low doses [50,51,79,80,81]. For example, researchers found that electron radiation positively affected the seed growth in barley (*Hordeum vulgare* L.), and after a series of doses, the 5 kGy maximal one induced the increase in the cell length and the cell width of the coleoptile [49]. The stimulatory effects of low levels of electron beam (15 kGy, 100 keV) radiation have also been reported by Doroshkevich, and according to their research, the irradiation stimulated various aspects of the plants’ development such as the germination rate, the germination capacity, and the height and weight of *Triticum aestivum* L. [48]. Some studies also established that EBTTX irradiation enhanced the functional properties of red radish seeds by increasing the content of carotenoid, chlorophyll, ascorbic acid, and total phenol at a low dose (<1 kGy), with slight inhibition of the germination process and a decrease in the yield [82].

Bacteria and pathogen stresses are known to have a negative effect on seed germination, plant growth, and post-harvest storage [48,83]. Researchers found that pretreating seeds with electron irradiation was very promising to alleviate the biotic stress. For instance, it significantly reduced the percentage of barley seed that was infested by *Helminthosporium sativum* (*synonymous with Drechslera teres*) after the treatment with 1, 2, 4, and 5 kGy irradiation, and it also slightly increased the seedlings’ weight at 1 kGy irradiation compared with that of the control [50]. After 7 kGy electron beam treatment for 1 min, the corresponding initial loads of *Salmonella* on contaminated tomato (*Solanum lycopersicum* L. esculentum) seeds and the *E. coli O157:H7* on lettuce seeds (*Lactuca sativa*) were clearly reduced by 4.36 and 2.21 log CFU/g, respectively. It seems that the sterilization effect of the treatments was more efficient against *Salmonella* than it was against *E. coli O157:H7*. In Chinese cabbage (*Brassica rapa* ssp. *Pekinensis*) seeds, 0–3 kGy electron treatments activated the defense mechanism to the pathogen (*Fusarium graminearum*) and reduced the infection rate, while the seed quality had not been reduced below the commercial threshold of 91.83% seed germination [80]. The stress resistance effects induced by low levels of electron beam radiation have also been reported by Waskow, and they showed that 8–60 kGy irradiation inactivated the microbial pathogens and stimulated various aspects of the plant’s development such as accelerating the germination of *Lens culinaris* seeds [47]. However, it must be mentioned that the specific impact on seeds also depends on the type of pathogen, the radiation source, and the seed used. In many cases, the electron beam either decreases the pathogen contamination on the seeds without reducing the germination or increases the germination and enhances the seedlings’ establishment while inactivating the microbial pathogens. It indicates that the electron beam has promise for food control and agricultural production applications [51,84,85].

### 2.4. Priming with Proton Radiation

Proton radiation, a kind of particle radiation, is an important part of solar particle events (approximately 87%), and it is composed of protons (hydrogen nuclei) with a certain energy (1~1000 MeV). When they are impinging on tissues, fast-moving protons deposit the densest energy at the end of their path, the characteristic map of the absorbed dose as a function of the penetration depth shows a maximum at the position before the particle stops (the “Bragg peak”) [38,40,86]. Proton radiation stimulates organisms in a non-thermal manner, and it can impact the organisms in direct and indirect ways by damaging their DNA or generating ROS in the cells [86]. It has been reported that the protons are usually absorbed by the plant, and they cause various physiological responses, including promoting the water absorption of seeds, increasing the photosynthetic pigments, upregulating the ROS level, the plant hormones, and the antioxidants, accelerating the production of secondary metabolites, as well as enhancing the plant’s defense systems to strengthen crop production [38,54,87]. Several studies have been performed to investigate the effect of proton radiation on seed germination and seedling vigor. The experiment which exposed the seeds of soybean (*Glycine max* L. Merr.) with a 57 MeV proton beam revealed that the treatment increased the germination rate, promoted the accumulation of MDA, APX, chlorophyll, and together, it reduced the SOD and POD activities [55,88]. The pretreatment of rice seeds with low doses of proton beam irradiation (50 and 100 Gy) had also been reported, it stimulated seedling growth and significantly increased the plants’ height and root length [52]. Similarly, Kumar found a growth-stimulating effect on rice seedlings caused by low doses (20–40 Gy) of irradiation [53]. In addition, Oprica found that a 3 Gy proton beam irradiation accelerated the barley seedling growth (*Hordeum vulgare* L.) under 100 mM NaCl; the mode of action of the proton irradiation was related to the accumulation of soluble proteins and CAT, which helped to protect the cell membrane, maintain an oxidative balance, and enhance the stress resistance [54].

### 2.5. Priming with Heavy-Ion Beam Radiation

Heavy ions, various atoms with an atomic number greater than two, such as helium, carbon, neon, silicon, and argon, can be accelerated into a beam of heavy ions with certain energy by large particle accelerators. The ion beam is divided into three types according to different energies: low energy ion beams (10–100 keV/u), intermediate energy ion beams (10–100 MeV/u), and high energy ion beams (>100 MeV/u). Heavy-ion beams have the unique physical properties, including energy deposition, an inverted dose distribution, small-angle scattering, and a large damage section [89]. In contrast to traditional radiation, such as X-rays or γ-rays (sparse ionizing radiation), heavy-ion beams usually cause local and complex damage to genomic DNA molecules. As a dense IR, it has higher linear energy transfer (LET), which is defined as the energy per unit track length, and it shows a higher relative biological effectiveness in lethality and mutation [58,90]. Based on these, heavy-ion beams show many outstanding advantages, for instance, a lower damage rate, a higher mutation rate, and a wider mutational spectrum, which generate the unique variations which are different from those induced by electromagnetic radiation [91,92]. Further, ROS generated by ionizing the water molecules in cells during heavy-ion beam irradiation show a biphasic dose response, and on the one hand, it activates a variety of positive physiological and metabolic behaviors, such as breaking dormancy, accelerating germination, and enhancing the antioxidant capacity at low doses, while on the other hand, it causes negative oxidative stress and exerts inhibitory effects on organisms at high doses [25,93].

Several positive effects of the carbon ion beam on *Arabidopsis* seed have been reported by Wang, for example, radiation with a dose rate of 80 MeV/u at 50 Gy exhibited optimal stimulatory effects on the germination index, the root length, and the fresh weight of seedlings during germination, and they showed a higher stress tolerance to cold and heat compared with the non-treated samples. Interestingly, the irradiation enhanced the activities of SOD, POD, and CAT and increased the content of AsA and GSH in the seedlings, while it reduced the generation rate of O_2_^.−^, OH^.^, and H_2_O_2_ in the seedlings. Further, it also improved the accumulation of proline and soluble proteins, and it up-regulated the genes expression levels associated with cold and heat stress responses in the seedlings [24,25,56]. Pre-sowing with heavy-ions irradiation has been proved to perform stimulatory effects on seed germination as well as seedling growth. Ling reported that carbon ion beam irradiation (10 Gy) seed priming technology obviously increased the height, root length, and fresh weight of rice, together with the accumulation of the total soluble protein [57]. The stimulation effects of low levels of heavy-ion beam radiation have also been reported by Arena, and according to their study, 25 Gy Ca ions irradiation induced positive outcomes on the plants’ growth, such as a more compact plant size, larger berries, along with them being richer in carotenoids, ascorbic acid, and anthocyanins [94]. Rice seeds treated by argon (10 Gy) and carbon ions (10 Gy) also produced better seedlings sprouts, and the former one performed better in stimulating growth than the latter did [58]. Similarly, alfalfa (*Medicago sativa* L.) treated with a low-dose carbon ion beam (200 Gy) had a markedly improved germination rate and seed vigor [59]. Moreover, irradiation also has a positive effect on the production of functional compounds. For instance, in *Dolichos melanophthalmus*, two doses (1 and 10 Gy) of a carbon and titanium ion treatment increased the contents of starch, H_2_O_2_, and AsA compared with those of the control [95]. A low-energy nitrogen ion beam (4.0 × 10^16^ ions/cm^2^) treatment at the pre-sowing stage also has had a stimulation impact on the content of soluble protein and GSH and the activities of CAT in wheat seedlings [96]. So, it is not difficult to conclude that seed priming with a low-dose heavy-ion beam could be considered to be a beneficial application in agricultural production.

## 3. Ionizing Physical Seed Priming and the Underlying Mechanisms

It has been well evidenced that many kinds of ionizing irradiations have the potential to be effective and valuable tools for a seed pre-sowing treatment. How to use appropriate ionization physical priming technology to exert hormesis effects, accelerate the rate of seed germination, enhance the vigor index and the seedlings’ quality, and ultimately improve the crop yield has attracted more and more people’s attention. To date, the physiological and molecular mechanisms involved in the hormesis of ionizing irradiation have not been comprehensively revealed, but some ideas about the internal factors in the plant have been presented.

### 3.1. Mechanism behind the Interaction between X-ray Radiation and the Plant Systems

X-rays have a wide range of positive effects on plants, and although the research about the application of X-rays in plant stimulation has mainly been conducted in the last century, the effect of low-dose radiation-induced DNA damage remains a subject of interest. The primary damage caused by the X-rays is considered to arise from the electrons generated in a cascade (on the time scale of tens of femtoseconds) after the interaction between the X-rays and the matter via absorption and inelastic scattering [97]. The theoretical basis on how the X-rays impact the plants suggests that the irradiation causes DNA damage (mismatches, breaks, and cross-links), chromosome aberration, and the excessive production of ROS, leading to the damage of proteins, lipid membranes, and nucleic acids, affecting the growth and physiological parameters, and ultimately, the crop yield [37,98,99]. The response of the plants to the X-rays depends on the radiation quality (e.g., the doses and dose rate), the plant characteristics (e.g., the species and genome size), and the external conditions for growth (e.g., temperature and water) [40,41,100,101]. The effects that the X-rays have at elevated doses are to a large degree, harmful and hindering, however, some research indicates that low irradiation doses positively affect the growth of the plant and its productivity [41,42,102,103]. Under low-dose treatments, X-ray irradiation promotes the plants to synthesize enzymatic and non-enzymatic products effectively to remove excess ROS, including SOD, CAT, APX, POD, AsA, and GSH. At the same time, by increasing the content of proline and total soluble proteins to regulate the osmotic pressure and protect the cell membrane, the secondary metabolites also play an essential role in stimulatory effect, and they help in developing leaves that can withstand irradiation and form a more compact habitus [37,40]. Meanwhile, X-rays also upregulate the content of polymerase and enhance the recovery strategies (i.e., gene excision repair mechanisms) in crops [42]. Further, the studies in vitro also reported that X-rays induced the redox modifications (reduction or oxidation) of redox-active cofactors in proteins, for example, the X-rays mainly damaged PSII by reducing the Mn centers to MnII5−8 and breaking the Mn di-μ-oxo units [97,104]. The current research about the specific mechanism of the X-rays’ impact on plants are mainly focused on a few aspects of the plant’s physiology, but our actual understand is still limited. New molecular and physiological mechanisms underlying the use of X-rays for seed priming need to be unveiled in the future.

### 3.2. Mechanism behind the Interaction between γ-ray Radiation and the Plant Systems

Nowadays, it is popular to use a low dose of γ-ray irradiation for priming the seeds or seedlings. This irradiation could efficiently accelerate seed germination and the seedling’s growth, enhance their tolerance to biotic or abiotic stress, which are conducive to improving the crop yield. Similar to other types of ionizing irradiation, the stimulatory or inhibitory effects on the plant’s growth depend on the seed species and status, the dose and dose rate, as well as exposure time. In general, the hormesis effect occurs during low-dose irradiation, while on the contrary, the inhibitory effect occurs at higher doses because of the over-produced free radicals damaging the physiological and biochemical properties of the plants [70,105,106,107]. According to the research, γ-ray radiation, as a high-energy type of IR, can penetrate the cell membranes and interact with tissues, producing ROS by ionizing the water molecules. It has been opined that ROS are a key regulator of the response to γ-rays, and they impact lipid peroxidation, oxidize and modify the proteins and nucleic acids, and they also act as secondary signal molecules to induce the associated defense signaling, subsequently triggering antioxidant responses. To alleviate the damage caused by salinity, heat, and heavy metals, γ-ray irradiation reduces the ROS content in the plants by improving the activities of the antioxidant enzymes, including SOD, CAT, POD, and APX, together with the synthesis of the substance-protecting cell membranes and the balancing of the osmotic pressure, for instance, proline and soluble proteins [46,68,75,108,109]. These results provide a substantial theoretical basis for promoting seed initiation, seedling growth, and overcoming the daily stress factors.

The genome-wide expression profiling analysis reveals that the ROS is accepted by the ROS receptors after being generated by water radiolysis. Subsequently, oxidative signal-inducible kinase (OX) is regulated by phosphoinositide-dependent kinase (PDK) or the signaling molecular Ca^2+^, and after that, OX will regulate the transcription factors (TFs) by changing into mitogen-activated protein kinase (MAPK) to further amplify the defense signaling after irradiation. These TFs include the heat shock factor (HSF), LTP family protein (LTPs), zinc finger protein (Zat), apetala-1 (AP-1), WRKY DNA-binding protein (WRKY), NAC family (NAC), the regulator of the atpase of the vacuolar membrane (RAV), Myb domain protein (Myb), and others. The research showed that γ-ray irradiation upregulated the expression level of the genes associated with ROS scavenging, such as CAT3 (At1g20620), Blue copper binding protein (At5g20230), Ferritin1 (At5g01600), and AOX putative (At1g32350), and it promoted the metabolisms associated with scavenging surplus free radicals to maintain the optimal redox status in plants [110,111]. Similarly, the transcriptome analysis also showed that the growth stimulation of barley by low-dose γ-ray irradiation (20 Gy) involves the transcriptional control of the genes related to enhancing the antioxidant system as well as reducing the H_2_O_2_ levels, and the abundant proteins and cell wall components [112]. Further, in another study, γ-ray irradiation induces the expression of the genes associated with sucrose-starch metabolism, resulting in the significant accumulation of sugar and starch, which accelerates the accumulation of plant energy substances [113].

**Figure 5:**
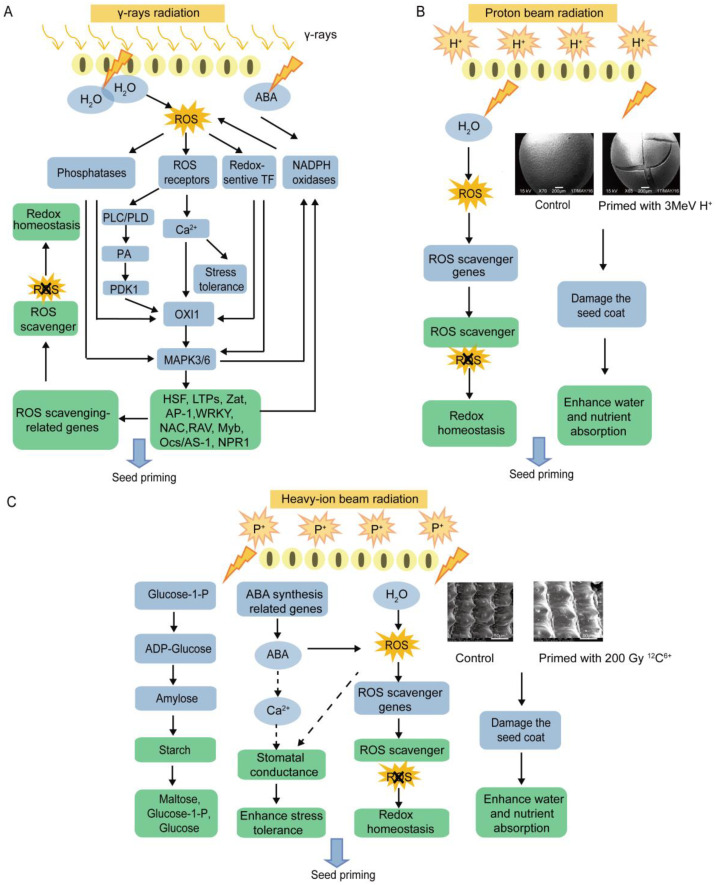
(**A**) Effect of γ-rays on seed germination, the ROS is accepted by ROS receptors after being generated by water radiolysis. Subsequently, oxidative signal-inducible kinase (OX) is regulated by phosphoinositide-dependent kinase (PDK) or the signaling molecular Ca^2+^, and after that, OX will regulate transcription factors (TFs) by changing into mitogen-activated protein kinase (MAPK) to further amplify the defense signaling after irradiation. These TFs include heat shock factor (HSF), LTP family protein (LTPs), zinc finger protein (Zat), apetala-1 (AP-1), WRKY DNA-binding protein (WRKY), NAC family (NAC), the regulator of the atpase of the vacuolar membrane (RAV), Myb domain protein (Myb), and others. As a consequence, the genes associated with ROS scavenging and metabolites associated with scavenging surplus free radicals are produced to maintain the optimal redox status in plants. In addition, γ-ray irradiation upregulates the related genes to accumulate abscisic acid (ABA), leading to the synthesis of ROS and the enhancement of stress tolerance. The figure is partially adapted from [110]. (**B**) Effect of proton beam on the coat and redox statuses of seed. Proton beam penetrates the seed coat, which it causes perforation and enhances water absorption and nutrient, meanwhile, it increases the content of antioxidants to scavenge ROS. (**C**) Effect of heavy-ion beam induction on seed germination. Heavy-ion beams induced the expression of genes associated with sucrose-starch metabolism, resulting in the significant accumulation of sugar and starch, which accelerated the accumulation of plant energy substances, and while it also generates ROS by water radiolysis, the ROS also acts as secondary signal molecules to further amplify the associated defense signaling, the redox homeostasis is regulated by the enhanced antioxidant enzyme system. In addition, it upregulates the related genes to accumulate ABA, and the endogenous ABA and ROS may further control the stomatal to mediate gas exchange and increase stress tolerance. It also penetrates the seed coat and causes perforation and enhance water absorption and nutrient. Particle (P^+^). Dotted lines show indirect regulation. Figure data is partially adapted from JQ Wang, WJ Li, and LX Yu’s work, which are unpublished data.

Biotic stresses such as viral or fungal infections, or abiotic stress such as irradiation, salinity, heat, and cold always accelerates inner abscisic acid (ABA) accumulation in plants [107,114]. As it supposed that this technology will alleviate the stress effects of the plants, it has been perceived that γ-ray irradiation could accumulate abscisic acid (ABA) by upregulating the related genes, and such a generation of plant hormones would lead to the synthesis of ROS, and in return, the antioxidant enzyme system would be enhanced by the over-production of ROS which would maintain the optimal redox status in the plants. Meanwhile, the ROS also act as secondary signal molecules to further amplify the associated defense signaling [29]. This point is also supported by transcriptomic data that the indicate that the plant stimulatory effects are involved in the ABA signaling pathway [112]. The endogenous ABA and ROS may further control the stomata to mediate gas exchange and increase stress tolerance [114]. Then, it was found that stomatal conductance was markedly increased by γ-ray irradiation in garden pea (*Pisum sativum* L), spinach (*Spinacia oleracea*), and okra (*Abelmoschus esculentus*) [105]. A representative figure detailing the mode of action carried out by γ-rays priming is given in Figure 5A.

### 3.3. Mechanism behind the Interaction between the Electron Beam Radiation and Plant Systems

As a relatively new technology, many researchers are involved in investigating the priming effect of electron irradiation in different species, however, its application is quite limited compared to a considerable number of studies on the impacts of other ionizing irradiation on organisms which have been carried out globally [3]. The exact mechanisms or biochemical processes by which electron irradiation affects seed priming are poorly understood to date. The electron beam is produced with a linear accelerator by accelerating electrons to a near speed of light, and its action principle is similar to γ-ray irradiation technology. Both of the methods use low-energy or high-energy rays to act on DNA directly, or ionized water to generate free radicals to indirectly act on macromolecular substances, consequently changing the genetic characteristics of the organisms, prime seeds, or killing the insect eggs and microorganisms [83]. Low-dose IR can promote seed germination, while the germination rate will gradually decrease or lag when it exceeds a certain radiation threshold [29]. The positive biological effect triggered by electron beam irradiation is usually involved in changing the functional products, for instance, decreasing the content of chlorophyll, promoting the synthesis of phenol, and disturbing the starch and sucrose metabolism [115]. Irradiating seeds or sprouts with an electron beam is also a promising approach for reducing the number of microbes and pathogens and producing safe and pathogen-free sprouts for humans. The studies show that the electrons are accelerated almost to the speed of light, and subsequently, they pass microbes and pathogens through the matrix resulting in the inactivation of the bacteria [80,84]. Based on a former study, an electron beam with a beam energy of 0.7 MeV has a penetration depth of approximately 2.3 mm, and thereby lower doses are needed for sterilizing the pathogens on broccoli seeds (0.55–1.17 kGy) with a diameter of approximately 1.8 mm and on red radish seeds (1.20–1.85 kGy) with a diameter of 2.8 mm. Further, the electron beam treatment presents more effective sterilization in *Salmonella typhimurium* than in it does in *Escherichia coli O157:H7*, and it is more useful in sprouts than it is in seeds [51,84]. It is assumed that low-dose irradiation increases the content of metabolites, activates the antioxidant enzymes, enhances biotic stress tolerance, which are consequently beneficial for the plant’s growth.

### 3.4. Mechanism behind the Interaction between Proton Radiation and the Plant Systems

Being an important physical component of space radiation, proton radiation has been broadly investigated in recent years, and it has a wide range of positive effects on seeds. Due to the character of the “Bragg peak’’, proton radiation is destined to have a superior biological effect in organisms because the densest energy is deposited at the end of the path [86]. It has been reported that the absorption of proton irradiation by organisms may directly cause the DNA double-strand to break and cause cluster lesions, subsequently triggering NHEJ to repair the damage [116,117]. In addition, proton irradiation triggers an oxidative imbalance and imposes stress on the seeds, and at the same time, it also stimulates the accumulation of antioxidants and proline to eliminate excess ROS and regulate the osmotic potential in the plants, resulting in the improvement of their stress resistance [54,55,114]. For example, Oprica found the ability of barley to resist salinity stress which was obtained by a proton irradiation treatment was involved in controlling a series of antioxidants [54]. Further, the transcriptome research revealed the underlying mechanism of the proton treatment was involved in the up/down-regulated numerous genes relevant to the plant’s defense, photosynthesis, plant hormones, and transcription factors (TF), and proton-beam irradiation had a distinct mutation spectrum and a gene expression pattern compared to that of the γ-ray treatment [87]. In addition, proton irradiation could easily penetrate the coat of the caryopses and cause the perforation of the outer tissue layers, and this creation of micropores facilitates water absorption and enhances the hydration of seeds, which is conducive to the first step of germination [38]. The research also demonstrated that the thickness of the seeds’ coat and the seeds’ size were relevant to the proton irradiation priming effect, for example, since 1 MeV ions were not able to penetrate the seed’s coat (≈40 ± 5 µm thickness), it did not affect the seed germination. The seed’s radiative resistance increases with the seed’s coat thickness and the seed’s size, but it decreases with the water content, therefore, the seed’s size and water content should be considered to be a potential selection criterion for seed priming by proton irradiation [38,118]. A representative figure detailing the mode of action carried out by proton beam priming is given in Figure 5B.

### 3.5. Mechanism behind the Interaction between Heavy-Ion Beam Radiation and the Plant Systems

Although heavy-ion beam irradiation, as a kind of physical mutagen, is widely used for breeding, and it is more efficient than other physical methods are, such as X-rays, γ-rays, and electron beams, its application in terms of seed priming has been ignored. The interaction between the heavy ion beams and the organisms involves not only energy deposition but also mass deposition and strong ionization along the track in the process of irradiation, abundant free radicals, and more intense energy level transitions will be produced, leading to the breaking of chemical bonds and resulting in a relatively high and complex biological effect. Unlike low-LET radiation, i.e., X-rays, γ-rays, and electron beams, which mainly induce DNA single-strand breaks and single-base damage, the damage triggered by the heavy-ion beam is more prone to double-strand breaks, cluster lesions, and large base deletions. In addition, although the heavy ion beam and proton have a unique depth–dose curve, forming a Bragg peak, this peak becomes narrower with an increasing atomic number, and the relative dose increases. Therefore, it exhibits higher RBE and LET than proton irradiation does [89,92,119,120,121,122,123,124,125].

According to previous studies, similar to a proton treatment, the technical principle of the heavy-ion beam is also involved in imposing mechanical pressure and an etching effect on the seed’s coat, causing damage and perforation to the surface of the receptor cells, and enhancing the cell membrane permeability [126,127,128,129]. Therefore, it may cause the alteration of the seed’s characteristics and facilitate the water absorption of the seeds, it may and further accelerate the hydration and germination. In addition, the mode of action of heavy-ion beam irradiation is likely associated with the accumulation of antioxidants, which scavenge the ROS to maintain an oxidative balance, which is conducive to helping the plant to overcome the irradiation stress [25,93]. By performing a transcriptome analysis, Ya revealed that the positive rice seed priming effect caused by a low-energy nitrogen-ion beam was associated with the upregulation of the signaling proteins, kinases, plant hormones, transcription factors, secondary metabolites, resistance proteins, peroxidase, and chromatin modification [130]. The accumulation of endogenous ABA has been established to enhance the stress tolerance and regulated the synthesis of ROS in the cell, which act as the signal molecular to amplify the plant’s defense response under irradiation and participate in seed priming [29,130,131]. Further, in another study, heavy-ion beam irradiation induced the expression of the genes correlated to sucrose-starch metabolism to significantly accumulate sugar and starch, which accelerated the accumulation of the plant’s energy substances [113]. A representative figure detailing the mode of action carried out by heavy-ion beam priming is given in Figure 5C. The mechanism of seed initiation by heavy ion beam needs to be further studied.

## 4. Conclusions and Future Aspects

To meet the demand of the agricultural market for better quality seeds and to fulfill the food need of the increasing world population, seed priming technologies have become more and more essential since many negative factors such as climate change, land pollution, and seed embryonic or shell development are still great challenges for seed germination. Therefore, some of the more time-saving, efficient, as well as greener for the environment methods are needed for seed priming and agricultural production. IR could be a promising alternative strategy for agricultural science and food technology as scientists have found that it curtails the germination time, enhances the seedling establishment, and augments the biochemical index associated with stress tolerance and improves the plants’ growth to achieve a better agronomic performance, further, it could also ensure that the seeds have a better physiological or phytosanitary quality by directly killing the microorganisms on the seeds’ surface. Seed invigoration by IR would be an difficult but unique alternative to the traditional methods.

Previous studies have reported that the mechanism underlying seed priming with ionizing irradiation are mostly involved in altering the physiological and biochemical molecular gene expression, as well as the morpho-structure. Additionally, the biological effects of radiation on the plants are strongly correlated to the dose, dose rate, duration of exposure, genome size, and species. However, the further micro mechanisms, and the correlation between the above reported factors, as well as the radiation response hotspots are still unknown. Therefore, the mechanisms of radiation-induced seed priming should be investigated for further commercial applications. In the future, the integration of the molecular and omics technologies will provide a better understanding of the mechanisms of irradiation priming. For instance, on the one hand, the micro response of the seeds to an IR agent and the molecular regulatory networks will be comprehensively revealed by combining the genome-wide, transcriptome, metabolome, and proteome data. However, on the other hand, matrix-assisted laser desorption/ionization time-of-flight imaging mass spectrometry will help us to visualize the differences in the distribution of the metabolites in real-time during the germination process.

Finally, it must be mentioned that the ionizing physical technique has the potential to be an effective approach to optimize seed priming and the agricultural yields to achieve global food security. However, the further application of this technique depends on a variety of factors related to (i) establishing the ionizing irradiation facilities that are able to work at an industrial scale, and (ii) establishing an irradiation reference system of seed priming for efficient application to more species.

## Figures and Tables

**Table 1:** Main types of ionizing radiation and their characteristics.

Type of Radiation	Discoverer, Year of Discovery	Wavelength (nm)	Frequency (EHz)	LET, keV/μm	RBE	Types of Ionizing Radiation
X-ray	W.C. Röntgen (1895)	0.01–10	0.03–30	<3.5	1	Electromagnetic waves
Gamma-ray	P.U. Villard (1900)	<0.01	>30	<3.5	1	Electromagnetic waves
Electron	Ernst Wagner (1948)	–	–	<3.5	1	Electron
Proton	K. P. Jackson (1970)	–	–	0.23–4.6	1–3	Protons
Heavy-ion beam	E.O. Lawrence (1930)	–	–	22.5–4000	1–10	Particles

LET: Linear energy transfer, RBE: Relative biological effectiveness.

**Table 2:** Summary of plant responses upon seed priming with ionizing physical treatments.

Treatments	Plant Species	Intensity/Dosage	Description of Responses	References
X-ray radiation	*Hibiscus esculentus* L.	0, 0.25, 0.5, 1, 2.5, 5, 10, 25, 50, 75, and 100 Gy at a dose rate of 1.9 kGy/min	Increased in plant height and weight, and total pigment, enhanced the activities of enzymatic antioxidants as well as increased the accumulation of nonenzymatic antioxidants	[37]
	*Brassica oleracea* L., *Pastinaca sativa* L.	2 and 8 Gy at a dose rate of 16.7 mGy/s	Advanced the first emergence counts of broccoli (*Brassica oleracea* L.) and parsnip (*Pastinaca sativa* L.)	[39]
	*Solanum lycopersicum* L.	0.3, 10, 20, 50, and 100 Gy at a dose rate of 1 Gy/min	Increased the plant height, number of leaves, and plant leaf area, and formed more compact plants	[40]
	*Coffea arabica*	0, 50, 100, 150, 200, and 400 Gy at a dose rate of 8 Gy/s	Improved germination, as well as enhanced seedling vigor and the hypocotyl growth	[41]
	*Phaseolus vulgaris*	0.3, 10, 50, and 100 Gy at a dose rate of 1 Gy/min	Significantly stimulated leaf lamina growth, mildly increased the activity of poly (ADP-ribose) polymerase (PARP), together with an over-production of phenolic compounds in cells	[42]
γ-ray radiation	*Holoptelea integrifolia, Oroxylum indicum*, *Terminalia chebula*	25, 50, 100, 150, 200, 250, and 300 Gy at dose rates of 1.386 kGy/h	Accelerated seed germination	[35]
	*Lathyrus chrysanthus* Boiss	0, 50, 100, 150, 200, and 250 Gy at dose rates of 0.8 kGy/ h	Increased germination percentage, seedling and root lengths, fresh weight, dry matter content, as well as the total chlorophyll content	[36]
	*Hordeum vulgare* L.	0, 2, 4, 6, 8,10, 13,16, 20, 25, and 50 Gy at dose rates of 20, 60, and 350 Gy/h	Accelerated the seedling development, along with higher contents of glucose-6-phosphate dehydrogenase, pyruvate kinase, and guaiacol peroxidase activity	[43]
	*Vicia sativa* L.	100 Gy at a dose rate of 354 Gy/h	Alleviated the deterrent from salt and drought stress with enhancement of the activities of catalase (CAT), superoxide dismutase (SOD), and ascorbate peroxidase (APX) and accumulated proline contents and increased dry weight	[44]
	*Pennisetum gluucum* L.	0, 0.25, 0.5, 0.75, 1.0, and 2.0 kGy with a dose rate of 33 Gy/min.	Significantly reduced the percentage of fungal incidence without changing the final germination capacity	[45]
	*Hordeum vulgare* L.	0, 50, 100, 150, 200, 250, and 300 Gy at a dose rate of 6.25 Gy/min.	Enhanced the tolerance to lead and cadmium stress with reduction of hydrogen peroxide (H_2_O_2_) and malondialdehyde (MDA) contents, enhancement the activities of antioxidant enzyme and proline levels, and alteration in chloroplasts ultrastructure	[46]
Electron radiation	*Lens culinaris*	180 kV; 0, 8, 16, 32, and 60 kGy	Accelerated seed germination, caused root abnormalities, inactivated microbial pathogens	[47]
	*Triticum aestivum* L.	100 and 130 keV; 3 and 15 kGy	Increased germination, plant height and weight, reduced fungi infection	[48]
	*Hordeum vulgare* L.	1 MeV; 500, 1000, 1250, 1500, 1750, 2000, 3000, 4000, 5000, 6000, 7000, 8000, 9000, 10,000, and 1100 Gy	Increased in coleoptile elongation, cell length, and cell width	[49]
	*Hordeum vulgare* L.	1, 2, 3, 4, 5, 6, 7, and 8 kGy a dose rate of 500 Gy/imp	Increased germination, reduced the disease incidence	[50]
	*Solanum lycopersicum* L. *esculentum*	150 keV; 7 kGy	Reduced the initial load of pathogenic bacteria	[51]
Proton radiation	*Brassica rapa*	1, 2, and 3 MeV with ion fluence 10^13^ ions cm^−2^	Penetrated the seed coat and caused perforation to promotes germination	[38]
	*Oryza sativa* L.	45 MeV; 0, 50, 100, 200, 300, 400, 500, 600, 700, and 800 Gy	Increased in plant height and root length	[52]
	*Oryza sativa* L.	14.52 MeV; 20, 40, 60, 80, 100, 120, 150, 180, 200, 220, 250, 280, 300, 350, 400, 450, 500, 550, and 600 Gy	Increased in plant height, shoot and root length	[53]
	*Hordeum vulgare* L.	150 MeV; 0, 3, and 5 Gy	Improved seedling growth, and enhanced salinity stress tolerance	[54]
	*Glycine max* L. Merr.	57 and 100 MeV; 55, 62, 110, 117, 168, 172, 243, 246, 316, and 308 Gy	Increased the germination rate, but reduced the survival rates	[55]
Heavy-ion beam radiation	*Arabidopsis thaliana*	0, 50, 100, 150, and 200 Gy at dose rate of 80 MeV/u	Increased the germination index, root length, and fresh weight, increased the generation rates of superoxide anion radical (O_2_^.−^ ), hydroxyl radical (OH^.^), and H_2_O_2_, along with enhancing the activities of SOD, peroxidase (POD), CAT, ascorbate (AsA), and glutathione (GSH)	[25]
	*Arabidopsis thaliana*	0, 50, 100, 150, and 200 Gy at dose rate of 80 MeV/u	Higher stress tolerance to cold, decreased the generation rate of O_2_^.−^, OH^.^, H_2_O_2_, and MDA, enhanced accumulation of enzyme and non-enzymatic antioxidant, and upregulated the expression levels of cold-regulated genes	[24]
	*Arabidopsis thaliana*	0, 50, 100, 150, and 200 Gy at dose rate of 80 MeV/u	Higher stress tolerance to heat, reduced the generation rate O_2_^.−^, OH^.^, H_2_O_2_, and MDA, enhanced enzyme activities and non-enzymatic antioxidant content, and up-regulated the genes expression level associated with heat stress response	[56]
	*Oryza sativa* L.	320 MeV; 0, 20, 40, 60, 80, 100, and 120 Gy	Increased plant height, and fresh weight, along with the total soluble protein content	[57]
	*Oryza sativa* L.	10 Gy	Improved seedling growth	[58]
	*Medicago sativa* L.	0, 200, 400, 800, and 1200 Gy	Enhanced seed germination and vigor	[59]

Gy: gray, kGy: Kilogray, mGy: milligray, s: second, min: minute, h: hour, imp: impulse, u: nucleon, keV: kiloelectron volt, MeV: Million electron volts.

## Data Availability

Data is presented in manuscript.
